# Association of Prophylactic Corticosteroids with Post-Extubation Outcomes in Pediatric Cardiac Critical Care: A Retrospective Propensity-Weighted Cohort Study

**DOI:** 10.3390/jcm15124762

**Published:** 2026-06-18

**Authors:** Kwannapas Saengsin, Noraworn Jirattikanwong, Pakpoom Wongyikul, Phichayut Phinyo, Thirasak Borisuthipandit, Rekwan Sittiwangkul, Suchaya Silvilairat, Krit Makonkawkeyoon, Saviga Sethasathien, Tin Ayurag, Nateewit Wiwatkamonchai, Kanokkarn Sunkonkit

**Affiliations:** 1 Division of Cardiology, Department of Pediatrics, Faculty of Medicine, Chiang Mai University, Chiang Mai 50200, Thailand; kwannapas.saengsin@cmu.ac.th (K.S.); rekwan.s@cmu.ac.th (R.S.); suchaya.s@cmu.ac.th (S.S.); krit.makon@cmu.ac.th (K.M.); saviga.seth@cmu.ac.th (S.S.); 2 Center for Clinical Epidemiology and Clinical Statistics, Department of Biomedical Informatics and Clinical Epidemiology (BioCE), Faculty of Medicine, Chiang Mai University, Chiang Mai 50200, Thailand; noraworn_j@cmu.ac.th (N.J.); pakpoom.w@cmu.ac.th (P.W.); phichayut.phinyo@cmu.ac.th (P.P.); 3 Division of Pulmonology and Critical Care, Department of Pediatrics, Faculty of Medicine, Chiang Mai University, Chiang Mai 50200, Thailand; thirasak.b@cmu.ac.th; 4 Faculty of Medicine, Chiang Mai University, Chiang Mai 50200, Thailand; tin_ayurag@cmu.ac.th (T.A.); nateewit_w@cmu.ac.th (N.W.); 5 Division of Pulmonology and Sleep Medicine, Department of Pediatrics, Faculty of Medicine, Chiang Mai University, Chiang Mai 50200, Thailand

**Keywords:** post-extubation stridor, corticosteroids, extubation failure, pediatric cardiac intensive care unit

## Abstract

**Background/Objectives**: Post-extubation stridor (PES) is common in pediatric critical care and may contribute to extubation failure, particularly in children with heart disease. Prophylactic corticosteroids are frequently used before extubation, but their benefit in pediatric cardiac patients remains uncertain. We evaluated the association of prophylactic corticosteroids with PES and extubation failure and explored whether PES mediated any association with failure. **Methods:** We performed a retrospective, single-center, observational cohort study of extubation events in a pediatric cardiac critical care unit from July 2016 to June 2024. Exposure was prophylactic intravenous corticosteroids before planned extubation, most commonly dexamethasone (0.15–0.5 mg/kg per dose) or methylprednisolone (1–2 mg/kg per dose), administered 6–24 h before extubation in single- or multi-dose regimens. The primary outcome was clinically defined PES; the secondary outcome was extubation failure, defined as reintubation within 48 h. Confounding was addressed using propensity scores with inverse-probability weighting after common-support restriction. Causal interpretation of the weighted and mediation estimates was considered conditional on the no-unmeasured-confounding (ignorability) assumption. Subgroup analyses were stratified by PES status, and exploratory mediation analysis used structural equation modeling. **Results:** Among 494 extubation events, prophylactic corticosteroid use was not associated with lower odds of PES after weighting (OR 1.06, 95% CI 0.53–2.10) or extubation failure (OR 0.49, 95% CI 0.19–1.24). Among patients with PES, corticosteroid use was associated with a non-significant reduction in extubation failure (OR 0.70, 95% CI 0.14–3.43). Exploratory mediation analysis, interpreted under the ignorability assumption, did not support PES as a meaningful mediator. **Conclusions:** In this single-center cohort, prophylactic corticosteroid use was not associated with reduced PES or extubation failure. The findings do not support clinically defined PES as a key mediator of any potential treatment effect. Prospective studies are required for confirmation.

## 1. Introduction

Post-extubation stridor (PES) is a common complication in pediatric patients with congenital or acquired heart disease who undergo mechanical ventilation. It results from upper airway edema and inflammation, often leading to increased work of breathing, hypoxemia, and respiratory distress. In severe cases, it may progress to extubation failure and require reintubation [1,2]. Reintubation is associated with adverse outcomes, including prolonged intensive care unit (ICU) stay, increased morbidity, and higher healthcare costs [1,2,3]. Therefore, identifying effective strategies to reduce the incidence of PES may help improve extubation outcomes and reduce downstream adverse events.

Prophylactic corticosteroids are among the most commonly used strategies to reduce airway inflammation and prevent PES in critically ill children [4,5]. Several studies have suggested that administering corticosteroids before extubation reduces extubation failure [6,7]. This benefit is believed to occur through the prevention of PES, as adult trials and meta-analyses have demonstrated that multi-dose steroid regimens reduce post-extubation laryngeal edema and the need for reintubation in high-risk patients [5,8].

However, evidence confirming that prophylactic steroids effectively prevent PES in pediatric patients is insufficient, particularly in cardiac populations [9,10,11]. Moreover, it remains unclear whether any observed reductions in extubation failure are mediated specifically through reductions in PES. Pediatric patients with congenital or acquired heart disease represent a particularly vulnerable population with respect to extubation-related airway complications. Beyond the general risks associated with mechanical ventilation, these children are exposed to additional pathophysiological stressors, including cardiopulmonary bypass-related systemic inflammation, prolonged ventilatory support, fluid overload with capillary leak, repeated airway instrumentation, and postoperative laryngeal dysfunction. These factors increase the risk of post-extubation airway obstruction and subsequent extubation failure [12].

Given that pediatric cardiac patients may develop PES or extubation failure through distinct mechanisms. Therefore, as preliminary evidence, this study aims to (1) examine the causal association of prophylactic corticosteroids with PES and extubation failure in pediatric cardiac critical care and (2) evaluate whether any observed association between corticosteroid use and extubation failure is mediated through PES using real-world data.

## 2. Materials and Methods

### 2.1. Study Design and Population

This study was conducted using a retrospective observational cohort design, encompassing retrospective data from July 2016 to June 2024. The data related to every extubation event of patients admitted to the Pediatric Cardiac Critical Care Unit (PCICU) of Chiang Mai University Hospital were gathered and collected. This study was approved by the Institutional Review Board of the Faculty of Medicine, Chiang Mai University (Approval No. 204/2025; 22 May 2025). Data were accessed for research purposes on 1 June 2025. The study was conducted in accordance with the Declaration of Helsinki (2024). [13] Informed consent was waived due to retrospective design. Owing to the minimal-risk design and use of fully anonymized data, the requirement for informed consent was waived.

During manuscript preparation, the authors used ChatGPT (OpenAI, version 5.0) solely to refine language, improve clarity of expression, and correct grammar. The tool was not used for study design, data analysis, interpretation of findings, or generation of scientific content. All scientific content, conclusions, and final wording were reviewed, revised, and approved by the authors.

### 2.2. Treatment and Outcome Definitions

Treatment:

Prophylactic corticosteroid use was defined as the administration of intravenous corticosteroids within 24 h prior to planned extubation, in accordance with hospital protocol. The most commonly used agents were dexamethasone and methylprednisolone. Dexamethasone was administered at doses ranging from 0.15 to 0.5 mg/kg per dose, whereas methylprednisolone was given at 1 to 2 mg/kg per dose. The number of doses varied from a single dose to multiple doses (commonly 2–4 doses) depending on the clinical situation.

Primary Outcome:

Post-extubation stridor (PES), defined as the presence of inspiratory stridor occurring within 6–24 h after extubation and ascertained solely on the basis of documentation in the medical record by the primary physician.

Secondary Outcomes:

Extubation failure is defined as the need for reintubation within 48 h after the extubation attempt.

The study included all eligible extubation events during the study period. Because some children underwent more than one extubation, the unit of analysis was the extubation event rather than the individual patient.

### 2.3. Statistical Analysis

#### 2.3.1. Descriptive Statistics

All statistical analyses were performed using Stata 17 (StataCorp, Lakeway, TX, USA). Descriptive statistics were used to summarize baseline characteristics. Categorical variables were presented as frequencies and percentages. Continuous variables were reported as means with standard deviations (SD) or medians with interquartile ranges (IQR), depending on their distributions. Comparisons between two groups were conducted using Fisher’s exact test for categorical variables. Student’s *t* test was used for the comparison of normally distributed continuous variables and the Mann–Whitney U test for the comparison of nonnormally distributed continuous variables.

#### 2.3.2. Methods to Minimize Confounding

This study used inverse probability of treatment weighting (IPTW) with weight stabilization (SW) to account for confounding. This method creates a pseudo-population in which treated and untreated participants are weighted by the inverse of their propensity scores (PS) and the inverse of one minus the PS, respectively [14]. The PS is defined as the probability of receiving prophylactic steroids based on baseline characteristics considered to be potential pretreatment confounders using multivariable logistic regression analysis utilizing fractional polynomials to model non-linear continuous predictors, while employing a robust clustered variance method with the individual patient identifier specified as the cluster variable to account for intra-group correlation [15,16,17]. A set of prespecified pretreatment confounders influencing steroid selection were included in the PS model: sex, age, body weight, duration of intubation more than 7 days, endotracheal tube with cuff, history of extubation failure, fluid balance status 24 h before extubation (positive or negative between input and output), having genetic disease, type of congenital heart (acyanosis, cyanosis with SpO_2_ < 85, and cyanosis with SpO_2_ ≥ 85), admit PCICU due to post-operation. Covariate balance both before and after weighting was assessed using the standardized difference (STD). Variables that remained imbalanced after SW (defined as STD > 0.1) were addressed through double adjustment by including these covariates in the outcome model [18]. To fulfill the positivity assumption, a common support region was defined as the overlapping range of propensity scores between treated and untreated patients, thereby avoiding zero probability of receiving certain exposure or mediator levels [19].

To account for extreme weight that may influence the estimation of treatment effect, we performed a post hoc sensitivity analysis using weight truncation, capping extreme stabilized weights at the 1st and 99th percentiles [19,20,21].

#### 2.3.3. Method for Handling Missing Data

Missing baseline data were handled using multiple imputation by chained equations under the missing-at-random assumption. Continuous variables were imputed using predictive mean matching with five nearest neighbors, whereas binary variables were imputed using logistic regression. The imputation model included all variables used in the causal analysis to ensure model compatibility and reduce the risk of biased estimates. The number of imputations was determined using a two-stage approach based on von Hippel’s quadratic rule to ensure stable and replicable standard error estimates [22]. Final estimates and standard errors were pooled using Rubin’s rules, which account for both within- and between-imputation variability [23].

#### 2.3.4. Outcome Analyses

**Figure 1:**
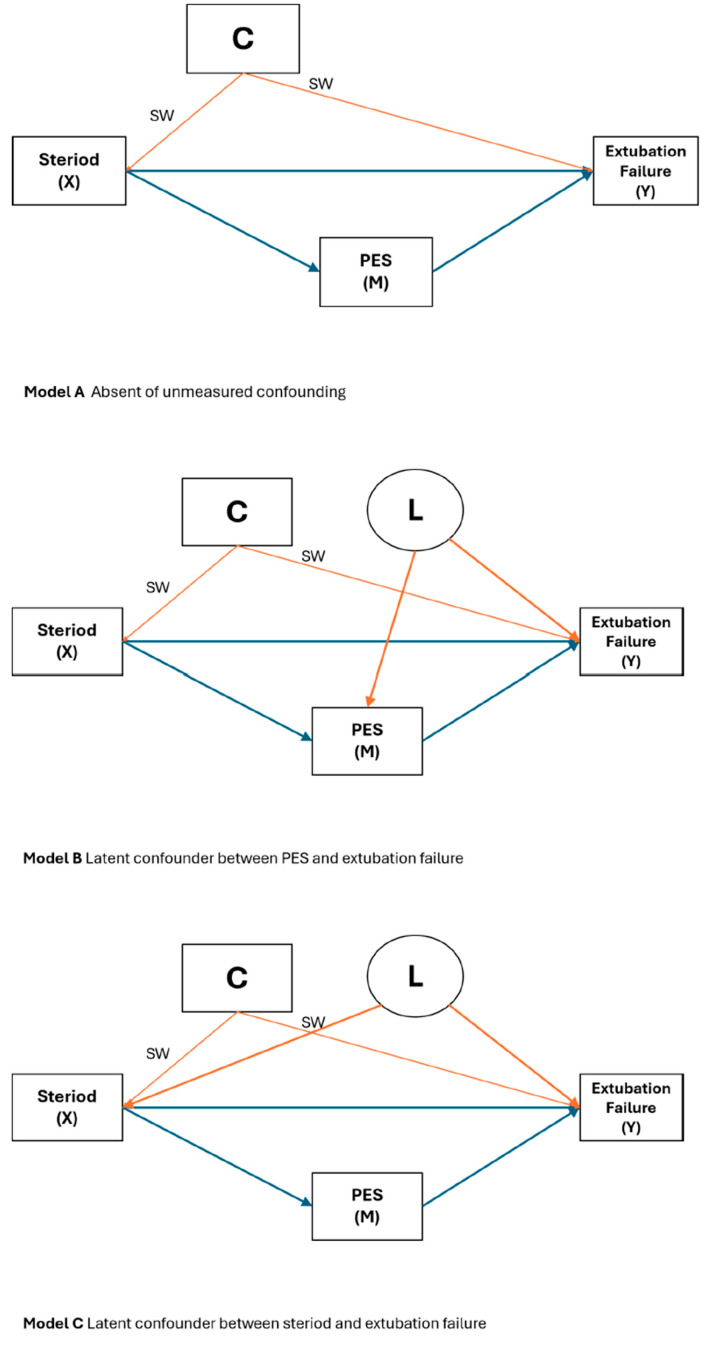
Hypothesized structural causal model. The blue line represents the main open causal pathway for primary and secondary outcomes. The orange line represents the open non-causal pathway with variables that vary across models.

Figure 1 illustrates the hypothesized causal structure (Model A). The average treatment effect of prophylactic steroids on PES and extubation failure, and mediation analysis were conducted using a generalized structural equation model (GSEM) [18,24]. To account for the intrinsic correlation between multiple events within the same patient, we utilized the robust clustered variance method, using the individual patient identifier as the cluster variable. Mediation analysis was conducted to determine whether PES mediates the effect of prophylactic steroids on extubation failure by decomposing the total effect (TE) into natural direct effect (NDE) and natural indirect effect (NIE). Estimation of the indirect and direct effects from data relies on the assumptions of no unmeasured confounding between intervention and mediator and between mediator and outcome, as well as no confounders between the mediator and outcome that are affected by the treatment, which we call the ignorability assumption [25].

Sensitivity analyses for ATE via unadjusted model, propensity score stratification and stabilized weight with weight truncation (at less than the 1st and more than the 99th percentile) method will be performed on each outcome. Furthermore, subgroup analyses stratified by PES status were conducted to evaluate the incremental predictive value of PES for treatment response with respect to extubation failure.

#### 2.3.5. Post Hoc Analysis

To address potential violations of the ignorability assumption, specifically unmeasured mediator-outcome confounding, we introduced a latent variable (L), representing unmeasured patient-level factors (e.g., subclinical airway reactivity or disease severity) that could simultaneously influence both PES and extubation failure. Following the structural framework for sensitivity analysis in causal mediation, we fixed the variance of L to 1 and iteratively varied the path coefficients from L to the mediator (L **→** M) and L to the outcome (L **→** Y) [Figure 1 Model B]. We examined the impact of these “hidden” paths on the NIE to determine the stability of our findings. This allowed us to assess whether a latent confounder of a plausible clinical magnitude could shift the NIE from a null or counter-intuitive direction into the hypothesized protective direction (OR < 1.0). A similar approach was applied in Model C, where unmeasured exposure-outcome confounding was introduced [Figure 1, Model C] to assess if such confounding could shift the Total Effect (TE) toward the null.

## 3. Results

### 3.1. Study Population

**Figure 2:**
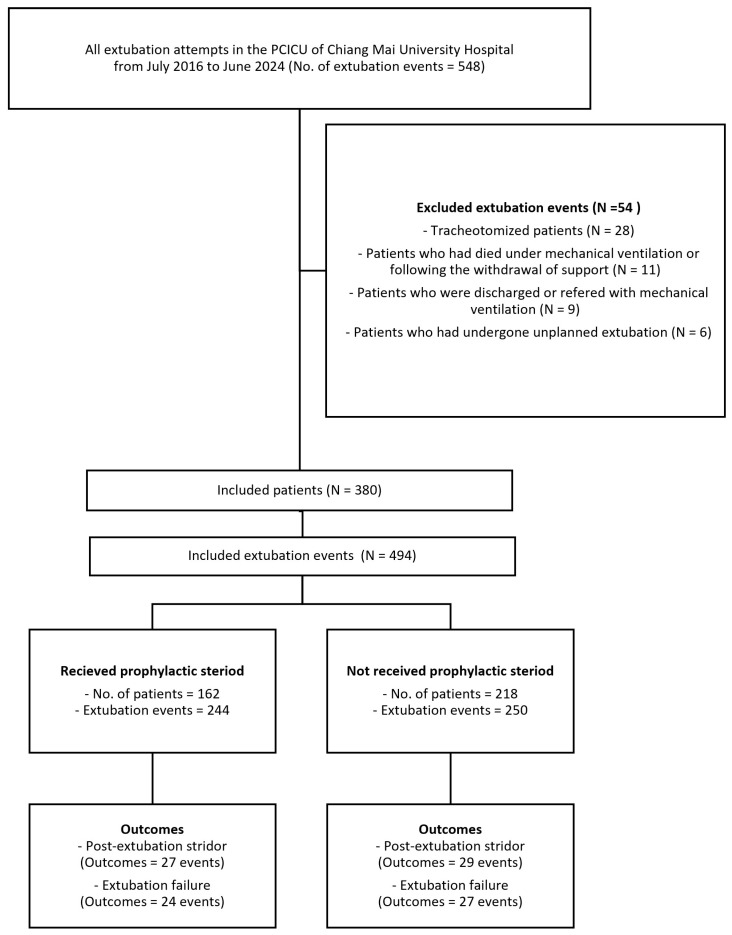
Study flow diagram.

The final cohort comprised 380 unique patients, with a total of 494 extubation events included in the analysis, comprising 244 events that received prophylactic corticosteroids and 250 events that did not (Figure 2). Seventy-two patients (18.9%) experienced at least one repeat extubation. The number of events per patient ranged from 1 to 5. Baseline covariates before propensity score weighting are summarized in Table 1. Patients receiving prophylactic corticosteroids were significantly male (STD = −0.120), younger (STD = −0.220), lower body weight (STD = −0.192), and more often experienced prolonged intubation (>7 days) (STD = 1.067), previous extubation failure (STD = 0.264), and ICU admission after major surgery (STD = 0.306), compared with those who did not receive steroids. Dexamethasone served as the primary corticosteroid, administered in 96.3% of cases with recorded agent data. Other steroids, such as methylprednisolone (2.3%), hydrocortisone (0.9%), and prednisolone (0.5%), were utilized rarely. Notably, specific agent information was unavailable for 12.3% of the total cohort.

### 3.2. Covariate Balance After Propensity Score Weighting

**Figure 3:**
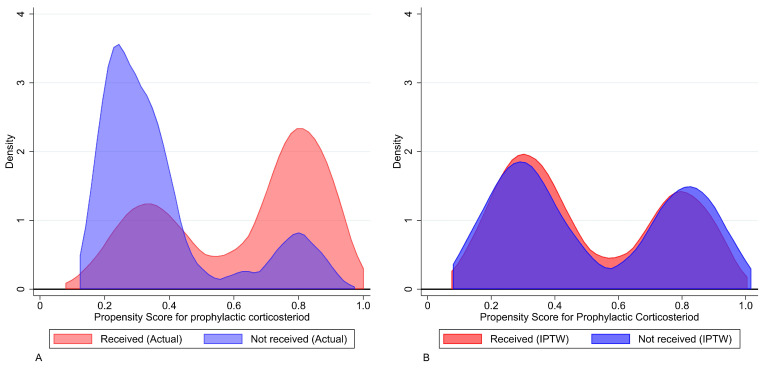
Propensity score distribution overlap plot for actual (**A**) and IPTW (**B**).

**Figure 4:**
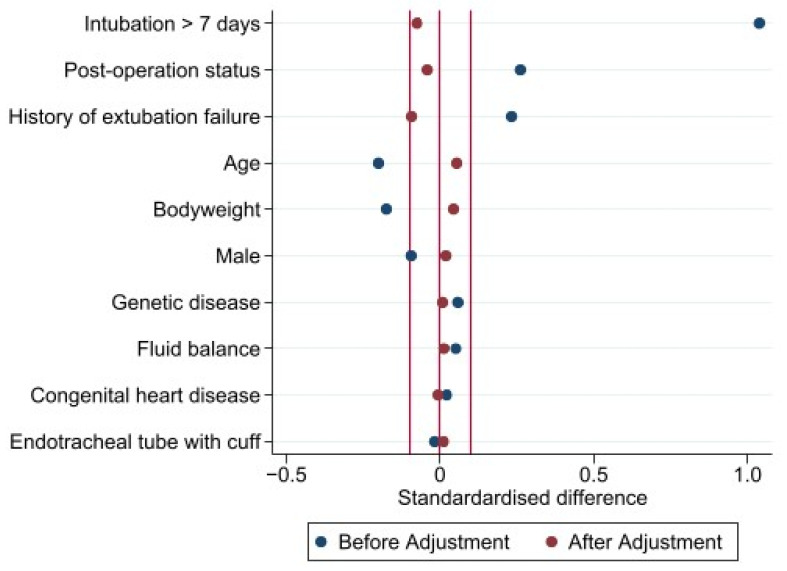
Standardized differences (STD) of baseline covariates between study groups before and after propensity score weighting.

Median and range of PS of patients receiving and not receiving prophylactic corticosteroids were 0.74 (0.15–0.95) and 0.30 (0.06–0.94), respectively (Figure 3). A total of 15 extubation events were excluded due to violation of the positivity assumption. After applying propensity score weighting within the common support region, all covariate balances were achieved with post-weighting STDs reduced to <0.1 (Figure 4). Details on the comparison between pre-weighting and post-weighting are provided in Table 2.

### 3.3. Effect of Prophylactic Corticosteroids on Post-Extubation Outcomes

The crude odds ratio (OR) of prophylactic corticosteroid provided no effect on both PES and extubation failure outcomes. After SW, prophylactic corticosteroid use was not associated with a reduction in clinically defined PES, with an OR of 1.06 (95% CI 0.53–2.10; *p* = 0.878). Likewise, corticosteroid use was not significantly associated with lower odds of extubation failure in the overall weighted cohort (OR 0.49, 95% CI 0.19–1.24; *p* = 0.132), although the point estimate suggested a possible protective effect.

### 3.4. Subgroup Analysis Stratified by Post-Extubation Stridor

**Figure 5:**
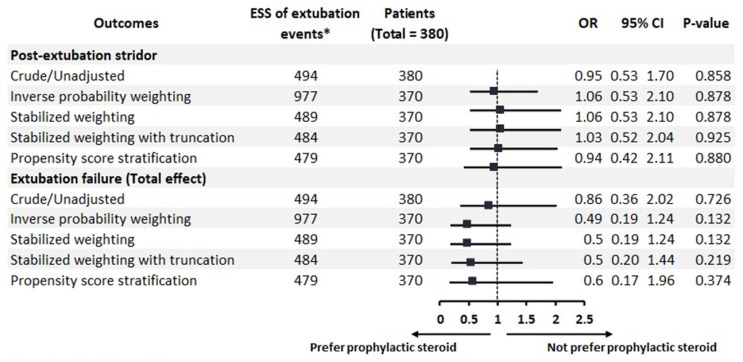
Forest plot of treatment effect; of the total 494 extubation events, 15 extubation events were excluded after common support trimming in both propensity methods to maintain the positivity assumption. * Effective sample size of extubation events. Abbreviations: ESS, effective sample size; OR, odds ratio; CI, confidence interval.

Table 3 presents the association between prophylactic corticosteroid use and extubation failure stratified by PES status. Among patients who developed PES, a subgroup comprising 54 extubation events, prophylactic corticosteroid use was associated with lower odds of extubation failure; however, this association was not statistically significant (OR 0.70, 95% CI 0.14–3.43; *p* = 0.662). In contrast, among patients who did not develop PES, prophylactic corticosteroid use was associated with significantly lower odds of extubation failure (OR 0.42, 95% CI 0.19–0.94; *p* = 0.035). Sensitivity analyses using stabilized weighting with truncation and propensity score stratification yielded similar findings. Detailed treatment effect estimates across analytic approaches are presented in Figure 4 and Figure 5.

### 3.5. Mediation Analysis

The mediation analysis results are presented in Table 4. In the primary mediation model (Model A), the total effect of prophylactic corticosteroid use on extubation failure was not statistically significant (OR 0.49, 95% CI 0.19–1.24; *p* = 0.132). However, the natural direct effect was statistically significant (OR 0.46, 95% CI 0.22–0.96; *p* = 0.039), whereas the natural indirect effect through PES was not significant (OR 1.05, 95% CI 0.56–1.97; *p* = 0.877). Under the assumption of no unmeasured confounding, these findings did not support PES as a meaningful mediator of the association between prophylactic corticosteroid use and extubation failure.

### 3.6. Post Hoc Analysis

**Figure 6:**
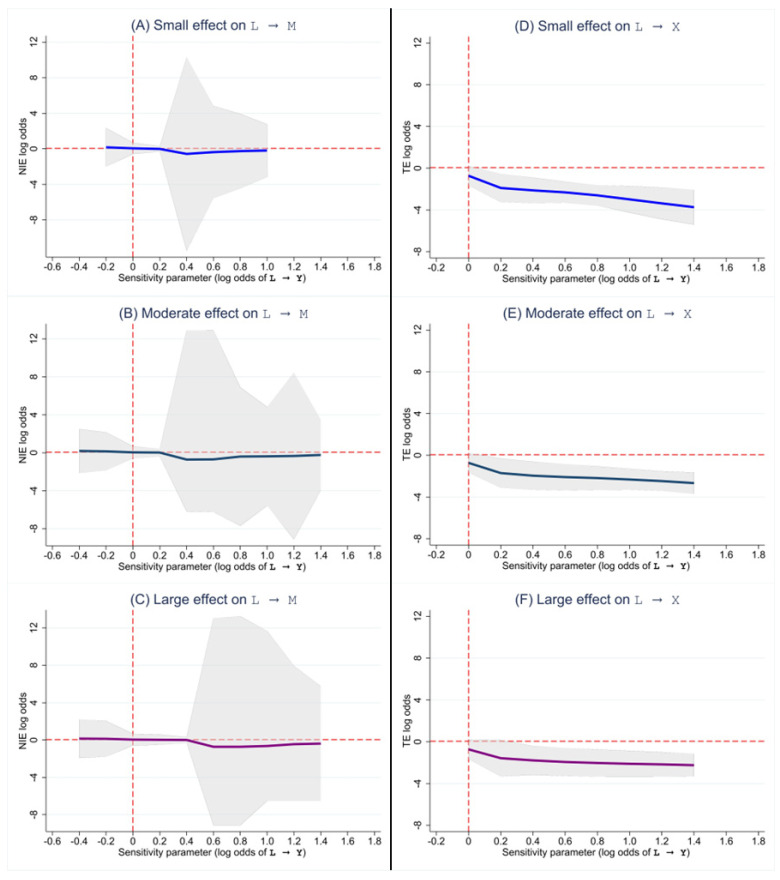
Post-hoc sensitivity analyses under Model B and Model C assumptions. The left panels (**A**–**C**) show changes in the natural indirect effect (NIE; log-odds scale) under Model B, while the right panels (**D**–**F**) show changes in the total effect (TE; log-odds scale) under Model C. Sensitivity analyses were performed by varying the assumed association between the latent confounder (L) and the outcome (Y), expressed as log odds. Three levels of association between the latent confounder and the mediator or treatment were examined: small (log odds = 0.3; panels (**A**) and (**D**)), moderate (log odds = 0.5; panels (**B**) and (**E**)), and large (log odds = 0.8; panels (**C**) and (**F**)). Solid lines represent point estimates, and shaded areas indicate 95% confidence intervals. Red dashed horizontal lines indicate the null effect (0 on the log-odds scale), and red dashed vertical lines indicate the primary analysis assumption of no unmeasured confounding (sensitivity parameter = 0). Abbreviations: NIE, natural indirect effect; TE, total effect.

Under Model B, which incorporated a latent unmeasured confounder affecting both the mediator and the outcome, sensitivity analyses were performed by varying the latent-variable paths. The effect of the latent variable on the mediator (L → M) was fixed at log-odds coefficients of 0.3, 0.5, and 0.8, while the effect on the outcome (L → Y) was varied from −0.4 to 1.4. As shown in Figure 6B, a hypothesized protective mediation effect (NIE < 0 on the log-odds scale) emerged only under assumptions of at least small-to-moderate unmeasured confounding. In contrast, the sensitivity analysis under Model C showed that the total effect remained robust across a range of assumptions regarding unmeasured exposure–outcome confounding, with treatment effect estimates remaining broadly consistent with the primary analysis (Figure 6C).

## 4. Discussion

In this retrospective cohort of pediatric patients with congenital or acquired heart disease admitted to a cardiac intensive care unit, we evaluated the association between prophylactic corticosteroid use and post-extubation outcomes using propensity score-based inverse probability weighting and exploratory mediation analysis within a causal inference framework. After adjustment for measured confounders, and conditional on the assumption of no unmeasured confounding, prophylactic corticosteroid use was not associated with a reduction in clinically defined PES. Although the overall association with extubation failure was not statistically significant, exploratory subgroup analyses suggested lower odds of extubation failure among patients who did not develop PES. Given the distinctive cardiopulmonary physiology and perioperative airway vulnerability of children with heart disease, the mechanisms underlying extubation outcomes in this population may differ from those assumed in general pediatric intensive care populations. Consistent with this possibility, under the assumptions required for mediation analysis, including sequential ignorability, mediation analysis did not support clinically defined PES as a meaningful mediator of the association between corticosteroid use and extubation failure. Sensitivity analyses suggested that the mediation findings may be influenced by departures from the ignorability assumption, whereas the overall pattern of estimated associations remained broadly consistent. These observations may be consistent with the possibility that any potential benefit of prophylactic corticosteroids may operate through alternative pathways, although any such interpretation remains conditional on the underlying causal assumptions, and such mechanisms were not directly assessed in the present study. As this study was conducted at a single tertiary pediatric cardiac referral center, the findings should be interpreted in the context of institution-specific perioperative management and extubation practices.

### 4.1. Effect of Prophylactic Corticosteroids on Extubation Failure

After propensity score weighting achieved balance in measured baseline covariates, the point estimate for prophylactic corticosteroid use suggested lower odds of extubation failure; however, the association did not reach statistical significance in the overall weighted analysis. A similar estimate was observed in the primary mediation model, in which the total effect remained non-significant. The consistency of these point estimates across analytic approaches suggests a potentially favorable association, although the precision of the estimates was limited. Accordingly, these findings should be interpreted cautiously and regarded as hypothesis-generating rather than confirmatory.

Notably, exploratory subgroup analysis showed that among patients who did not develop PES, prophylactic corticosteroid use was associated with lower odds of extubation failure, whereas no clear association was observed among those who developed PES. Although these subgroup findings should be interpreted with caution, they raise the possibility that any potential benefit of corticosteroids may not be mediated primarily through clinically apparent upper-airway obstruction. This interpretation is further supported by the mediation analysis, which did not identify clinically defined PES as a meaningful mediator of the association between corticosteroid use and extubation failure.

From a biological perspective, corticosteroids may plausibly influence extubation outcomes through several mechanisms beyond clinically evident stridor. Endotracheal instrumentation and peri-extubation airway manipulation can provoke mucosal inflammation, endothelial activation, and increased capillary permeability, while pediatric cardiac patients may also experience postoperative systemic inflammation, altered pulmonary mechanics, and fluid shifts that contribute to extubation vulnerability [5,12,26,27,28,29]. Glucocorticoids may attenuate these processes through genomic and non-genomic anti-inflammatory effects, stabilization of endothelial barrier function, reduction in tissue edema, and enhancement of epithelial fluid clearance [26,27,28,29]. In this context, any potential benefit may reflect broader effects on respiratory physiology or systemic inflammatory burden rather than a measurable reduction in clinically defined PES.

These findings are broadly consistent with prior studies suggesting that systemic corticosteroids may improve extubation-related outcomes in selected high-risk pediatric patients [4,6,27,30]. However, unlike some earlier reports, our study did not demonstrate a reduction in clinically defined PES, and the overall association with extubation failure remained statistically non-significant. Therefore, while the observed pattern of results is compatible with a possible clinical benefit, residual confounding, heterogeneity in corticosteroid regimens, and limited precision must be considered. Sensitivity analyses suggested that the overall treatment effect estimates were broadly stable across alternative analytic assumptions, although mediation-related inferences were more vulnerable to potential departures from ignorability assumptions. Given the observational design and the potential for confounding by indication, these results should be interpreted as supportive but not definitive evidence for targeted corticosteroid prophylaxis in high-risk pediatric cardiac patients.

### 4.2. Post-Extubation Stridor and Subgroup Findings

In contrast to extubation failure, prophylactic corticosteroid use was not associated with a lower adjusted incidence of clinically defined PES. This finding is broadly consistent with prior literature showing that the effect of corticosteroids on PES has been variable across heterogeneous intensive care populations, particularly in pediatric settings and unselected cohorts [12,29]. By contrast, several randomized trials and systematic reviews, especially in high-risk adults and selected pediatric subgroups, have reported reductions in post-extubation airway edema, stridor, and, in some cases, reintubation when corticosteroids are administered before planned extubation [4,5,12,28,30,31,32,33]. One possible explanation for the absence of an observed reduction in PES in our pediatric cardiac cohort is that post-extubation airway compromise in this population is likely multifactorial, with contributions from prolonged intubation-related laryngeal injury [26,27], fluid overload [32,33], postoperative systemic inflammation after cardiopulmonary bypass [34,35,36], and altered respiratory mechanics such as atelectasis, reduced compliance, and diaphragmatic dysfunction [37,38]. These factors may not be fully mitigated by corticosteroids alone.

Exploratory analyses stratified by PES status showed differing associations between corticosteroid use and extubation failure. Among children who developed PES, corticosteroid use was associated with a non-significant reduction in extubation failure. In contrast, among those who did not develop PES, corticosteroid use was associated with significantly lower odds of extubation failure. These findings should be interpreted cautiously, given the small PES-positive subgroup, the resulting imprecision of the estimates, and the fact that PES is a post-treatment variable that may lie on the causal pathway. Accordingly, these subgroup analyses are best considered exploratory and hypothesis-generating. Nonetheless, the observed pattern raises the possibility that any potential benefit of corticosteroids may involve broader respiratory or systemic effects beyond clinically apparent upper-airway obstruction.

### 4.3. Insights from Causal Mediation Analysis

A major strength of this study is the application of causal mediation analysis to examine whether the association between prophylactic corticosteroid use and extubation failure operates through clinically defined PES. In the primary mediation model, the natural indirect effect (NIE) through PES was not significant, indicating no evidence that clinically defined PES served as a meaningful mediator under the assumption of no unmeasured confounding. These findings suggest that any potential association between corticosteroid use and extubation outcomes may involve pathways not fully captured by overt PES, such as broader anti-inflammatory effects, improved lower-airway mechanics, or enhanced fluid clearance [5,12].

The post hoc sensitivity analysis further informed the interpretation of these findings. Under Model B, a protective indirect effect emerged only when assuming at least small-to-moderate unmeasured mediator–outcome confounding, such as latent disease severity or baseline airway reactivity. This pattern suggests that the mediation results are sensitive to departures from the ignorability assumption. By contrast, under Model C, the total effect (TE) estimates remained broadly consistent across a range of assumptions regarding unmeasured exposure–outcome confounding, supporting the relative stability of the overall treatment effect pattern. Nevertheless, because some attenuation was observed under latent-confounding scenarios, these findings should be interpreted cautiously.

Taken together, the current results raise the possibility that any potential benefit of prophylactic corticosteroids may operate through mechanisms other than clinically evident upper-airway obstruction. Such mechanisms may include attenuation of lower-airway or systemic inflammation, improvement in pulmonary mechanics and gas exchange, or acceleration of epithelial fluid clearance—processes that could facilitate successful liberation from mechanical ventilation even in the absence of overt stridor [39,40,41]. However, these pathways were not directly measured in the present study and should therefore be regarded as hypothesis-generating rather than established.

From a clinical perspective, the absence of a measurable reduction in clinically defined PES should not necessarily be interpreted as evidence of no benefit. Rather, the findings suggest that clinically apparent PES may be an incomplete surrogate for extubation success in pediatric cardiac patients. At the same time, given the observational design, limited precision of some estimates, and sensitivity of the mediation results to assumptions about unmeasured confounding, these interpretations warrant confirmation in larger prospective studies, including analyses within PES-defined subgroups [5,31,41].

### 4.4. Clinical Implications

These findings do not support a clear overall benefit of prophylactic corticosteroids with respect to extubation outcomes in this cohort, although the possibility of benefit in selected high-risk pediatric cardiac patients remains uncertain. Accordingly, PES alone may be an insufficient surrogate endpoint for extubation success in this population [6,30,31]. From a clinical perspective, any consideration of prophylactic corticosteroid use should be individualized and incorporated into a broader, risk-informed extubation strategy that includes optimization of fluid balance, cuff-leak assessment when feasible, standardized sedation weaning, and proactive post-extubation respiratory support [5,12,42,43]. Given that no reduction in clinically defined PES was observed, airway-focused preventive measures—such as appropriate endotracheal tube sizing, atraumatic airway management, and close post-extubation monitoring—should remain essential components of care regardless of corticosteroid use [5,12,42]. More broadly, this study highlights the value of applying causal inference methods to observational data in pediatric critical care, as such approaches may help distinguish overall treatment effects from intermediary pathways and support more mechanism-informed refinement of clinical protocols [30,31].

### 4.5. Strengths

Our study offers several methodological strengths. We applied rigorous confounding control using propensity score-based inverse probability weighting, restricted estimation to the region of common support, and verified post-weighting balance via STD. The propensity model incorporated prespecified covariates that capture key clinical determinants of steroid selection in pediatric cardiac intensive care, enhancing face validity and mitigating selection bias. Beyond estimating average treatment effects, we employed generalized structural equation modeling to decompose total effects into natural direct and indirect components, enabling formal mediation inference. Finally, we complemented the primary mediation analysis with latent-variable sensitivity analyses to probe the robustness of conclusions under plausible violations of sequential ignorability and potential unmeasured confounding.

### 4.6. Limitations

There are several limitations that warrant consideration in our study. First, this study was conducted using retrospective observational data. Although our primary analyses applied propensity score weighting with stabilized weights, this approach relies on the assumption of no unmeasured confounding, which may not be fully met in our retrospective setting. In addition, several potentially important effect modifiers—such as surgical complexity, duration of cardiopulmonary bypass, intraoperative course, and the level of postoperative cardiorespiratory support—were not fully captured or standardized in the dataset. Several other clinically relevant predictors were also unavailable in our retrospective dataset, including variables related to post-cardiopulmonary bypass inflammatory status and airway anatomy, such as subglottic diameter. The absence of these variables may have contributed to residual confounding. In sensitivity analyses that introduced a latent (unmeasured) confounder, the estimated mediation effect was attenuated under scenarios with a substantial latent confounder, suggesting that the assumptions may not hold. Accordingly, the estimated NIE should be interpreted with caution. Second, the relatively small number of patients with PES constrained subgroup analyses, yielding imprecise, wide confidence intervals. This study was conducted at a single tertiary pediatric cardiac referral center, which may limit the generalizability of the findings to other institutions with differing patient populations, perioperative management strategies, and extubation practices. Future multicenter studies incorporating detailed perioperative variables and standardized protocols are needed to confirm the transportability of these findings. Third, although we restricted the definition of PES to cases ascertained solely from inspiratory stridor explicitly documented in the medical record by the primary physician within 6–24 h after extubation to prevent overlap, measurement error remains possible due to the absence of a standardized protocol for assessing PES. Future studies incorporating more objective or standardized assessments of upper-airway obstruction, such as cuff-leak testing, airway ultrasound, or endoscopic evaluation, may help improve outcome ascertainment. Fourth, corticosteroid regimens were heterogeneous with respect to agent, cumulative dose, dosing frequency, and timing of administration. In our cohort, dexamethasone was the predominant corticosteroid, whereas methylprednisolone and other agents were used infrequently; therefore, the observed estimates largely reflect a heterogeneous exposure pattern dominated by dexamethasone rather than a direct comparison between corticosteroid agents. Although we provided a detailed summary of regimen characteristics, this variability may have attenuated treatment effects and limited the ability to detect regimen-specific or dose–response relationships. This heterogeneity may also have obscured potential differences in effectiveness between dexamethasone and methylprednisolone. Given the known time-dependent biological effects of corticosteroids, such heterogeneity in exposure may also influence clinical effectiveness. However, regimen-stratified analyses were not feasible due to limited sample size within subgroups and variability in treatment patterns. Finally, despite the observed association between prophylactic corticosteroid use and extubation failure, important clinical uncertainties remain. In particular, the optimal corticosteroid regimen including agent selection, dosing, timing, and duration has not been clearly established, especially in pediatric cardiac populations. In addition, there is no consensus on which patients are most likely to benefit from prophylactic corticosteroids. In current practice, treatment decisions are often guided by clinician judgment and perceived risk factors, such as prolonged intubation, prior extubation failure, or anticipated airway edema, rather than standardized criteria. This variability underscores the need for more precise risk stratification tools and prospective studies to identify patient subgroups that derive the greatest benefit, as well as to define evidence-based corticosteroid protocols that can be consistently applied across centers. Future multicenter trials or pragmatic studies incorporating standardized corticosteroid regimens and predefined high-risk criteria are warranted to reduce practice variability and improve the consistency of care.

To address these gaps, future work should include multicenter pragmatic randomized or stepped-wedge trials in high-risk pediatric cardiac cohorts to confirm causality and define optimal corticosteroid regimens; embed standardized airway assessments (e.g., cuff-leak measurements, airway ultrasound, and laryngoscopy when indicated) to refine PES phenotyping and detect subclinical edema; and evaluate harmonized extubation bundles with predefined co-interventions to reduce treatment heterogeneity and enhance reproducibility across centers. Where feasible, future studies should also evaluate agent-specific regimens, particularly dexamethasone versus methylprednisolone, to clarify whether differences in corticosteroid selection contribute to variation in post-extubation outcomes.

## 5. Conclusions

In this single-center, retrospective, propensity-weighted cohort of pediatric patients with congenital or acquired heart disease, prophylactic corticosteroid use was not associated with lower odds of extubation failure or clinically defined PES. Under the assumptions required for mediation analysis, including no unmeasured confounding and sequential ignorability, exploratory mediation analysis did not support clinically defined post-extubation stridor as a meaningful mediator of the association between corticosteroid use and extubation failure. Any causal interpretation of these findings is conditional on untestable assumptions, particularly the absence of unmeasured confounding; given the observational design and the potential for residual confounding, these findings should be interpreted cautiously and regarded as hypothesis-generating. Further prospective multicenter studies are warranted to confirm these findings and to better define optimal corticosteroid strategies in this population.

## Figures and Tables

**Table 1:** Baseline characteristics.

	Missing Data N (%)	Received Prophylactic Steroid (N = 244) *	Not Received Prophylactic Steroid (N = 250) *	Unweighted Standardized Difference (STD)
Male, *n* (%)	0 (0.0)	122 (50.0)	140 (56.0)	−0.120
Age (month), median (IQR)	0 (0.0)	8.8 (3.1, 18.1)	10.7 (3.8, 36.6)	−0.220
Weight (kg), median (IQR)	0 (0.0)	5.5 (3.7, 8.2)	6.4 (4.2, 10.8)	−0.192
Duration of intubation > 7 days, *n* (%)	0 (0.0)	152 (62.3)	39 (15.6)	1.067
Endotracheal tube with cuff, *n* (%)	2 (0.4)	83 (34.3)	91 (36.4)	−0.049
History of extubation failure, *n* (%)	0 (0.0)	30 (15.9)	12 (6.6)	0.264
Fluid balance (ml/kg) 24 h before extubation, median (IQR)	15 (3.0)	6.0 (−8.4, 17.0)	5.0 (−8.6, 20.5)	0.017
Genetic disease, *n* (%)	0 (0.0)	71 (29.1)	66 (26.4)	0.074
Type of congenital Heart, *n* (%)	0 (0.0)			0.019
Cyanosis (SpO_2_ < 85)		49 (20.1)	50 (20.0)	
Cyanosis (SpO_2_ ≥ 85)		32 (13.1)	30 (12.0)	
Acyanosis		163 (66.8)	170 (68.0)	
Post-operation requiring PCICU, *n* (%)	0 (0.0)	181 (74.2)	147 (58.8)	0.306

* Number of extubation events. Abbreviations: IQR, interquartile range; kg, kilogram; ml, milliliter; PCICU, Pediatric Cardiac Critical Care Unit; SpO_2_, oxygen saturation; STD, Standardized Difference.

**Table 2:** Standardized differences (STD) of baseline covariates between study groups before and after propensity score weighting.

	Unweighted Standardized Difference	Weighted Standardized Difference
Male	−0.120	0.020
Age (months)	−0.220	0.055
Bodyweight (kilograms)	−0.192	0.044
Duration of intubation > 7 days	1.067	−0.075
Endotracheal tube with cuff	−0.049	0.011
History of extubation failure	0.264	−0.092
Fluid balance	0.017	0.012
Genetic disease	0.074	0.008
Congenital Heart status	0.019	−0.006
Post-operation	0.306	−0.041

**Table 3:** Effect of prophylactic corticosteroids on post-extubation stridor and extubation failure after stabilized weighting (SW) and subgroup analysis.

Outcome	Extubation Events (N */ESS **)	Odds Ratio (95% CI)	Risk Difference (95% CI)	*p*-Value
Stabilized Weighting (SW) with Common Support				
Post-extubation stridor	479/489	1.06 (0.53, 2.10)	0.00 (−0.06, 0.07)	0.878
Extubation failure (TE)	479/489	0.49 (0.19, 1.24)	−0.08 (−0.17, 0.01)	0.132
- In patients with PES	54/50	0.70 (0.14, 3.43)	−0.06 (−0.35, 0.22)	0.662
- In patients without PES	425/439	0.42 (0.19, 0.94)	−0.08 (−0.18, 0.01)	0.035

* Of the total 494 extubation events, 2 and 13 extubation events in patients with PES and without PES, respectively, were excluded after common support trimming to maintain the positivity assumption. ** ESS: Effective Sample Size. Abbreviations: CI, confidence interval; PES, post-extubation stridor; TE, total effect.

**Table 4:** Mediation analysis of the total, direct, and indirect effects of prophylactic corticosteroids on extubation failure mediated by post-extubation stridor (PES).

Effect	Path	Odds Ratio (95% CI)	*p*-Value
**Model A (No Unmeasured Confounding)**			
Total effect	**NDE + NIE**	0.49 (0.19, 1.24)	0.132
NDE	**X** **→** **Y**	0.46 (0.22, 0.96)	0.039
NIE (Through PES)	**X** **→** **M** **→** **Y**	1.05 (0.56, 1.97)	0.877

Abbreviations: CI, confidence interval; NDE, natural direct effect; NIE, natural indirect effect; TE, total effect; PES, post-extubation stridor.

## Data Availability

The datasets used and/or analysed during the current study are available from the corresponding author on reasonable request.

## Abbreviations

ATE	Average treatment effect
CI	Confidence interval
ETT	Endotracheal tube
GSEM	Generalized structural equation model
kg	Kilogram
ICU	Intensive care unit
IPTW	Inverse probability treatment weighting
IQR	Inter-quartile range
MAR	Missing at random
MICE	Multiple imputation by chained equations
ml	Milliliter
NDE	Natural direct effect
NIE	Natural indirect effect
OR	Odds ratio
PCICU	Pediatric cardiac intensive care unit
PES	Post-extubation stridor
PS	Propensity scores
SD	Standard deviation
SpO_2_	Oxygen saturation
STD	Standardized differences
SW	Stabilized weighting
TE	Total effect
